# Associations Between Lifetime Stressor Exposure and Externalizing Problems in Youth at Risk for Huntington Disease

**DOI:** 10.1002/brb3.71329

**Published:** 2026-03-30

**Authors:** Katherine E. McDonell, Shuhei Shiino, Elizabeth M. Key, Louis DeLuna, Kelly H. Watson, Bruce E. Compas, George M. Slavich, Daniel O. Claassen

**Affiliations:** ^1^ Department of Neurology Vanderbilt University Medical Center Nashville USA; ^2^ Department of Psychology and Human Development Vanderbilt University Nashville USA; ^3^ Department of Psychiatry and Biobehavioral Sciences University of California Los Angeles USA

**Keywords:** adolescents, behavior, Huntington disease, life stress, mental health

## Abstract

**Introduction:**

Children face significant stressors while growing up in families impacted by Huntington disease (HD). However, the sources of these stressors and how they are related to psychological difficulties have not been well‐characterized in this population. In this study, we examined the lifetime stressors experienced by youth at risk for HD using a comprehensive measure of lifetime stressor exposure and investigated how these stressors relate to psychological difficulties.

**Methods:**

Participants included 94 adolescents ages 10–18 years, 40 of whom were at genetic risk for HD (*M*_age_ = 13.7, SD = 2.9) and 54 of whom were community controls (*M*_age_ = 12.6, SD = 2.2). All participants completed the Stress and Adversity Inventory and Youth Self‐Report. Bivariate correlations and linear regression analyses were performed to examine the relationships between stressors, demographic characteristics, and psychological difficulties.

**Results:**

Adolescents at risk for HD reported significantly more lifetime stressors than controls, including greater frequency and severity of both acute life events and chronic difficulties. In addition, at‐risk participants reported more stressors involving Treatment/Health and Role Change/Disruption. Whereas lifetime stressors were primarily related to internalizing problems and anxiety for controls, stressor exposure in at‐risk individuals was most prominently related to externalizing problems, including rule‐breaking behavior.

**Conclusion:**

These results demonstrate that youth at risk for HD experience significantly more lifetime stressor exposure than their peers and report greater externalizing problems when exposed to stress. These findings highlight the importance of assessing lifetime stressor exposure in this clinical population and suggest key differences in how at‐risk youth may respond to stress.

## Summary

Growing up in a family impacted by Huntington disease (HD) can be stressful for children. However, we do not currently have a good understanding of the types of stress that these children face or how these stressors may be related to psychological problems. In this study, we measured stress exposure in children from HD families and controls from the community using a detailed questionnaire. We found that children from HD families reported significantly higher stress exposure than control children. The most common types of stress they reported were related to treatment and health and changes in roles and responsibilities. We also found that children from HD families were more likely to report problematic behaviors, such as breaking rules, when exposed to stress. These results help us learn how to better support children from HD families.

## Graphical abstract

This study examined lifetime stressor exposure in adolescents at genetic risk for Huntington disease (HD) compared to community controls. At‐risk participants reported significantly greater lifetime stressor exposure than controls and were more likely to report externalizing problems such as rule‐breaking behavior when exposed to stress.

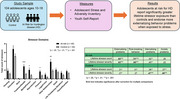

## 1 Introduction

Huntington disease (HD) is an autosomal dominant neurodegenerative disorder caused by a cytosine‐adenine‐guanine (CAG) repeat expansion in the HTT gene. HD is characterized by progressive cognitive, psychiatric, and motor impairment, typically beginning in early adulthood and progressing until death (Pagan et al., 2017). Children of affected individuals have a 50% chance of inheriting the causative gene expansion and thus developing the disease themselves. Individuals with HD and their family members are faced with significant stress over the course of the illness, both due to the relentless progression of the disease itself and the risk to future generations.

Many children from HD‐affected families grow up in uncertain and unstable environments, marked by high rates of family conflict, dysfunctional parenting, and adverse childhood events (Kjoelaas et al., 2020, 2022; Vamos et al., 2007). Children are frequently exposed to psychiatric and behavioral changes in affected parents (Kjoelaas et al., 2020) and suffer from social isolation and lack of support (Forrest Keenan et al., 2007). Many also find themselves taking on caregiving responsibilities from an early age (Dondanville et al., 2019), all while coming to terms with their own risk of developing the same disease (Baum et al., 1997; Brouwer‐Dudokdewit et al., 2002). Families as a whole also struggle with social and financial challenges (Rodríguez‐Santana et al., 2023; Vamos et al., 2007), early loss of employment (van der Zwaan et al., 2022), and escalating caregiver burden (Modrzejewska‐Zielonka et al., 2022; Youssov et al., 2022). Furthermore, people affected by HD experience significant psychological distress (Dale et al., 2022), have difficulties with emotion recognition and social cognition (Zarotti et al., 2025), and endorse feelings of stigma and reduced health‐related quality of life (Boileau et al., 2020; Carlozzi and Tulsky, 2013). A recent study showed that children raised in an HD family report significantly more childhood trauma, including emotional abuse, physical abuse, and both emotional and physical neglect (Maffi et al., 2025). These stressors can have detrimental consequences for young people in HD families, including long‐term insecure attachment (Van der Meer et al., 2006) and higher levels of psychiatric symptoms (Bilal et al., 2024; Downing et al., 2012; Snow et al., 2024; Watson et al., 2025). HD‐related stressors also negatively impact family relationships and communication between children and parents (Watson et al., 2021), and difficulty coping with these stressors is associated with increased negative emotions in children and their parents (Watson et al., 2023).

The pervasive and potentially harmful nature of stress exposure in children at risk for HD emphasizes the need for evidence‐based resources to support affected individuals and their families. However, current evidence regarding stress in HD is largely drawn from qualitative studies (Baum et al., 1997; Forrest Keenan et al., 2007; Kjoelaas et al., 2020, 2022), with a distinct lack of quantitative data. Moreover, mirroring the larger stress literature (Slavich, 2019), the quantitative studies that do exist rely primarily on brief checklist measures consisting of only a few items that do not fully capture the full spectrum of stressors in HD, particularly in early‐stage and at‐risk individuals (Downing et al., 2012; Snow et al., 2024). The gold standard in measuring stress exposure relies on interview‐based measures (Harkness and Monroe, 2016), which have yet to be applied in HD. Furthermore, with the exception of one recent study conducted in Italy (Maffi et al., 2025), most existing studies have not directly compared stressors experienced by individuals impacted by HD to a control sample, limiting our ability to distinguish unique sources of stress in HD.

Another critical gap is the limited understanding of stress exposure in at‐risk children and adolescents under the age of 18, who often bear the brunt of HD‐related stress and the resulting dysfunctional family dynamics. Recent evidence suggests that youth at risk for HD exhibit deficits in working memory, which are associated with reduced coping skills and increased anxiety and depression, making them especially vulnerable to the effects of stress (Ciriegio et al., 2020). Furthermore, adolescents and young adults with CAG repeat expansions exhibit impairments in executive function and working memory that are apparent as early as age 18 and continue to progress over time (Pfalzer et al., 2023). Therefore, a detailed understanding of stressors experienced in childhood and adolescence is urgently needed to develop targeted interventions to help at‐risk individuals learn how to cope with stress before the cognitive abilities that support these skills begin to decline.

Finally, although prior studies in HD have investigated associations between stressor exposure and internalizing symptoms, such as depression and anxiety, none have examined correlations with externalizing symptoms, such as impulsive and rule‐breaking behaviors, which can be particularly problematic in HD (McDonell et al., 2020; Watson et al., 2025). Both internalizing and externalizing problems tend to emerge during adolescence and provide a framework for understanding psychopathology across the developmental course (Ma et al., 2025; Willner et al., 2016). Internalizing problems encompass a range of internally focused symptoms, such as feelings of depression, anxiety, fear, or withdrawal, while externalizing problems refer to outwardly focused behaviors including aggression, attention problems, and rule‐breaking behaviors (Carragher et al., 2015). Both internalizing and externalizing problems have been described in adolescents at risk for HD (Watson et al., 2025), but correlations between stress exposure and these behavioral syndromes have not yet been investigated.

To address these gaps, we administered a quantitative assessment of lifetime stressor exposure and a well‐validated measure of psychological difficulties to a group of adolescents at risk for HD and to control adolescents recruited from the community. Using these measures, we sought to (a) characterize the specific types and characteristics of stressors experienced by at‐risk youth compared to controls and (b) examine how different types of stressors occurring across the life course are related to internalizing and externalizing problems in HD.

## 2 Materials and Methods

### 2.1 Study Design

We assessed self‐reported stressor exposure and psychological difficulties in at‐risk participants and controls and investigated associations between these variables using a cross‐sectional design.

### 2.2 Participants and Procedure

Participants included 40 adolescents at genetic risk for HD and 54 community controls. At‐risk participants were recruited through a Huntington's Disease Society of America Level 1 Center of Excellence in the Southeast. Potential participants were recruited through standard of care visits for parents and from a larger ongoing study assessing social connectedness and communication in families affected by HD. Controls were recruited through the Research Notification Distribution List, a free recruitment tool that sends study information to an email list of approximately 40,000 prospective participants consisting of university and medical center faculty and staff as well as regional community members who have expressed interest in learning about research opportunities.

Inclusion criteria for at‐risk participants included (a) being between the ages of 10–18 years; (b) having at least one parent or grandparent with genetically confirmed HD (>37 CAG repeats); (c) being able to read English, complete the study procedures, and provide written assent; and (d) having a parent who is willing and able to provide informed consent and complete caregiver assessments. Inclusion criteria for controls included (a) being between the ages of 10–18 years; (b) being able to read English, complete the study procedures, and provide written assent; and (c) having a parent willing and able to provide informed consent and complete caregiver assessments. Exclusion criteria included any physical or cognitive impairment that would preclude participants from being able to complete the study, unwillingness to participate, or inability to provide informed consent.

All participants under the age of 18 completed a written assent form, and parents provided informed consent. Participants who were 18 years old provided written informed consent. Participants were compensated with a gift card upon completion of the study. The study was reviewed and approved by the Institutional Review Board (IRB #220196).

### 2.3 Measures

#### 2.3.1 Demographic Information

Participants were asked to self‐report their age, gender, race, ethnicity, and zip code.

#### 2.3.2 Stressor Exposure

Lifetime stressor exposure was measured using the Stress and Adversity Inventory for Adolescents (Adolescent STRAIN), an NIMH‐RDoC‐recommended online system for assessing stress in youth ages 10–18 (Slavich et al., 2019). The Adolescent STRAIN is a computerized interview‐based measure that mimics a clinician‐administered interview but in an online, self‐report format. Questions are presented one at a time in colloquial, developmentally appropriate language, and a series of tailored follow‐up questions are asked to assess each endorsed stressor's frequency, severity, and duration. The Adolescent STRAIN was administered to all participants on a computer in a private research room.

The Adolescent STRAIN takes approximately 25 minutes to complete and assesses 75 stressors relevant for this age range, including 33 acute life events (e.g., deaths of relatives, job loss, and negative health event), and 42 chronic difficulties (e.g., persistent health, work, school, relationship, and financial problems). Branching logic is used to assess the severity, frequency, exposure timing, and duration of each stressor endorsed. Stressors are aggregated into six core subscales and are further categorized into 12 primary life domains (i.e., Housing, Education, Work, Treatment/Health, Marital/Partner, Reproduction, Financial, Legal/Crime, Other Relationships, Parent/Guardian, Death, Life‐Threatening Situations) and five core social‐psychological characteristics (i.e., Interpersonal Loss, Physical Danger, Humiliation, Entrapment, Role Change/Disruption). All items are designed to be appropriate for adolescents. For instance, the Marital/Partner domain focuses on problems in romantic relationships, including arguments with a partner or a serious break‐up. The Financial domain asks about family difficulties paying for basic necessities such as food and housing, school, or clubs or sports teams (Slavich et al., 2019). These variables are, in turn, used to compute 115 cumulative lifetime stressor exposure summary scores that provide a comprehensive overview of an individual's exposure to stressors over the life course.

Two additional questionnaires are included within the Adolescent STRAIN to assess general physical and mental health complaints over the past month. The Kessler‐6 (K‐6) is a 6‐item questionnaire that measures nonspecific psychological distress, such as feeling sad, nervous, or restless, on a scale from 1 (never) to 5 (very often) (Kessler et al., 2002). The Physical Health Questionnaire (PHQ) is a 14‐item questionnaire that assesses the frequency of somatic symptoms, such as headaches, stomach pain, or dizziness (Spence et al., 1987).

The Adolescent STRAIN has very good concurrent validity and outstanding predictive validity for a variety of relevant physical and mental health outcomes in adolescents (Burani et al., 2023; Gruhn et al., 2024; Slavich et al., 2019). Usability and acceptability have also been shown to be very high in this age group (Slavich et al., 2019).

#### 2.3.3 Psychological Difficulties

Psychological difficulties were assessed using the Youth Self‐Report (YSR) from the Achenbach System of Empirically Based Assessment (ASEBA) (Achenbach and Rescorla, 2001). The YSR is a well‐validated, empirically driven instrument that assesses emotional and behavioral problems in a developmentally appropriate context. This self‐report measure generates both empirically based scales and DSM‐V‐oriented scales. The present study focused on seven psychological scales to examine a broad range of symptoms: (1) Anxious/Depressed, (2) Withdrawn/Depressed, (3) Attention Problems, (4) Rule‐Breaking Behavior, (5) Aggressive Behavior, (6) Depressive Symptoms, and (7) Anxiety Problems. In addition, the three composites were examined to provide global assessments of these problems: (1) Internalizing Problems, (2) Externalizing Problems, and (3) Total Problems. Raw scores on each subscale are converted to *T*‐scores with a mean of 50 and a standard deviation of 10, derived from a large normative sample that is representative of the U.S. population. The ASEBA measures have excellent internal consistency, test‐retest reliability, and construct validity (Achenbach and Rescorla, 2001). The YSR was completed by all participants on paper, and responses were subsequently entered into the ASEBA database for analysis.

### 2.4 Sample Size Considerations

Based on our preliminary data showing moderate effect sizes (Cohen's *d* = 0.4–0.7) for differences on the STRAIN and ASEBA subscales of interest, we calculated that a sample size of 90 participants would yield 80% power to detect an effect size of *d* = 0.53 with *α* = 0.05.

### 2.5 Statistical Analyses

Descriptive statistics were calculated for demographic variables, including age, gender, ethnicity, and median income. The Shapiro–Wilk test was used to assess normality for continuous variables prior to conducting parametric analyses. Independent‐samples *t‐*tests were used to compare continuous variables, and Fisher's exact tests were used to compare categorical variables. Scores for each STRAIN and YSR subscale were compared between at‐risk and control participants using independent‐samples *t*‐tests. Associations between STRAIN scores and YSR subscales of interest were tested using bivariate Spearman correlations. The Bonferroni adjustment was used to correct for multiple comparisons. Fisher's *Z*‐tests were used to compare correlation coefficients between groups. Linear regression analyses were performed to examine the relationship between total stressor count and psychological difficulties. Age, gender, and income were included as covariates, and Group x STRAIN interaction terms were included to determine whether these associations differed based on HD risk status. Analyses were performed using SPSS version 27.

## 3 Results

### 3.1 Descriptive Statistics

Demographic data for HD at‐risk participants and controls are shown in Table 1. At‐risk participants (*n* = 40) were 62.5% female and had a mean age of 13.7 years (SD = 2.9). Community controls (*n* = 54) were 42.6% female and had a mean age of 12.6 years (SD = 2.2). Based on self‐reported race, 2.5% of at‐risk participants were Asian, 2.5% were more than one race, and 95% were White. Controls were 9.3% Asian, 11.1% Black, 5.6% more than one race, and 74.1% White. 5.6% of controls and 0% of at‐risk participants were Hispanic. The median income based on zip code was $69,832 for at‐risk individuals and $95,664 for controls.

**TABLE 1:** Sample characteristics.

Characteristic	At‐risk (*n* = 40)	Control (*n* = 54)	*p*‐value
Age, mean (*SD*), y	13.7 (2.9)	12.6 (2.2)	0.05
Gender, No. (%)			0.06
Female	25 (62.5)	23 (42.6)	
Male	15 (37.5)	32 (59.3)	
Race, No. (%)			**0.02** ^†^
Asian	1 (2.5)	5 (9.3)	
Black	0 (0)	6 (11.1)	
More than one race	1 (2.5)	3 (5.6)	
White	38 (95)	40 (74.1)	
Ethnicity, No. (%)			0.26
Hispanic	0 (0)	3 (5.6)	
Non‐Hispanic	40 (100)	51 (94.4)	
Median income, mean (SD), $	69,831.75 (17565.34)	95,664.78 (29749.14)	**< 0.001**

^†^ Bold text indicates significance after correction for multiple comparisons.

There were no significant differences in age [*t* (69) = 1.95, *p* = 0.05] or gender distribution (Fisher's exact test *p* = 0.06) between HD at‐risk participants and controls. Consistent with the global demographics of HD, a higher proportion of our at‐risk participants were White (Fisher's exact test *p* = 0.02). Estimated median income based on zip code was significantly lower in the at‐risk group compared with controls, *t*(88) = −5.26, *p* < 0.001.

### 3.2 Lifetime Stressors

All participants completed the Adolescent STRAIN without difficulty, with a mean time‐to‐completion of 27.4 min (SD = 12.3), comparable to the expected time of 25 min based on Slavich et al. (2019). No participants discontinued the interview, and no complaints were reported.

To characterize overall physical and mental health complaints in HD at‐risk individuals and controls, we first compared scores on the Physical Health Questionnaire (PHQ) and Kessler 6‐Item Psychological Distress Inventory (K‐6) (Table 2). At‐risk participants reported significantly higher mental health complaints (*p* = 0.002) compared to controls. We next compared scores on the STRAIN core subscales between groups (Table 2). After correction for multiple comparisons, at‐risk participants scored significantly higher than control participants on multiple subscales, including total lifetime count of stressors (*p* = 0.002), total lifetime severity of stressors (*p* = 0.002), total count of acute life events (*p* = 0.002), total severity of acute life events (*p* = 0.002), total severity of chronic difficulties (*p* = 0.002), total count of stressors in the past 12 months (*p* = 0.002), and total severity of stressors in the past 12 months (*p* = 0.001).

**TABLE 2:** **Lifetime stressor exposure**. Scores are compared between at‐risk participants and controls for physical and mental health complaints and the core subscales of the Adolescent Strain. After correcting for multiple comparisons, at‐risk participants scored significantly higher for mental health complaints, total count of lifetime stressors, total severity of lifetime stressors, count of acute life events, and severity of acute life events.

Subscale	Mean score (SD)		*t*‐statistic	*p*‐value
	**At‐risk** (*n* = 40)	**Control** (*n* = 54)		
**Physical and Mental Health Complaints**				
Physical health complaints (PHQ)	35.0 (13.2)	28.8 (10.8)	2.47	0.015
Mental health complaints (K‐6)	14.5 (5.4)	11.3 (4.2)	3.19	**0**.**002** ^†^
**STRAIN Core Subscales**				
Total lifetime count of stressors	27.4 (18.9)	16.0 (12.8)	3.29	**0.002**
Total lifetime severity of stressors	49.0 (39.9)	25.9 (21.5)	3.33	**0.002**
Total lifetime count of acute life events	16.3 (11.4)	9.3 (8.5)	3.27	**0.002**
Total lifetime count of chronic difficulties	11.0 (8.5)	6.6 (5.4)	2.87	0.006
Total lifetime severity of acute life events	20.6 (16.0)	11.5 (10.0)	3.17	**0**.**002**
Total lifetime severity of chronic difficulties	28.4 (24.9)	14.4 (13.0)	3.26	**0.002**
Total count of stressors in the past 12 months	7.3 (5.0)	4.3 (3.7)	3.22	**0.002**
Total severity of stressors in the past 12 months	21.1 (16.9)	10.9 (11.0)	3.34	**0.001**

^†^ Bold text indicates significance after correction for multiple comparisons.

Abbreviations: PHQ = Physical Health Questionnaire; K‐6 = Kessler 6‐Item Psychological Distress Inventory.

**FIGURE 1:**
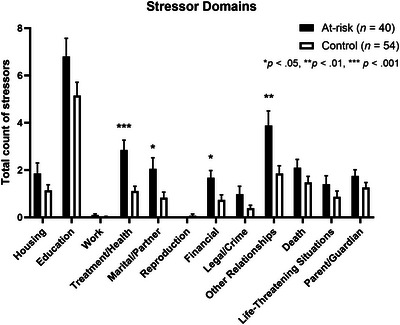
Total count of stressors across 12 primary life domains in at‐risk participants and controls. Bars show mean values; error bars indicate standard error of the mean (SEM).

**FIGURE 2:**
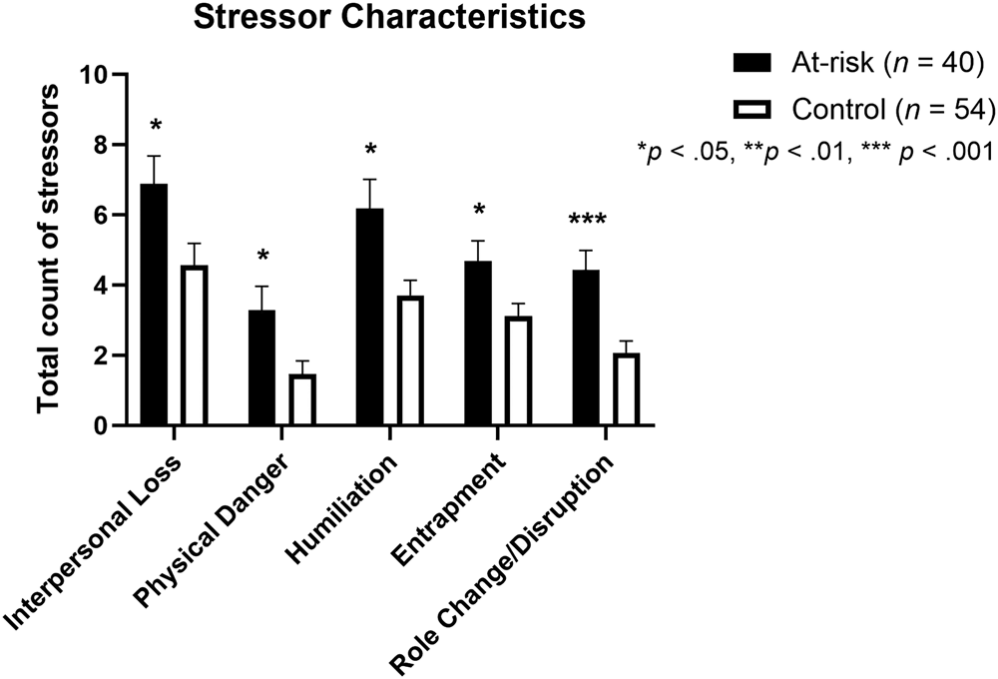
Total count of stressors across five core social‐psychological characteristics in at‐risk participants and controls. Bars show mean values; error bars indicate standard error of the mean (SEM).

To examine more specific sources of stress in HD at‐risk participants versus controls, we next compared scores on each of the stressor domains (Figure 1) and characteristics (Figure 2). After correction for multiple comparisons, at‐risk participants reported significantly more stressors involving Treatment/Health (*p* < 0.001) and Role Change/Disruption (*p* = 0.001). Controls did not score higher on any of the stressor domains or characteristics assessed by the STRAIN.

### 3.3 Psychological Difficulties

We next compared Youth Self‐Report scores between at‐risk participants and controls to assess psychological difficulties in each cohort (Table 3). At‐risk participants scored higher on the attention problems subscale (*p* = 0.009) and the total problems composite scale (*p* = 0.026). Although these differences were not significant after correcting for multiple comparisons, they were associated with moderate effect sizes (Cohen's *d =* 0.56 for attention problems and 0.47 for total problems), indicating that these differences may become more apparent with a larger sample size and greater statistical power. Controls did not score higher on any subscales.

**TABLE 3:** **Youth Self‐Report**. Scores on YSR symptom scales and composite scales are compared between groups.

YSR scale	Mean *T*‐score (SD)		*t*‐statistic	*p*‐value
	**HD** (*n* = 40)	**HC** (*n* = 54)		
**Symptom Scale**				
Anxious/Depressed	58.4 (7.7)	55.8 (6.9)	1.73	0.09
Withdrawn/Depressed	58.1 (7.8)	56.1 (8.0)	1.21	0.23
Attention problems	62.3 (8.9)	57.7 (8.0)	2.66	0.009
Rule‐breaking behavior	54.4 (6.1)	53.0 (4.1)	1.29	0.20
Aggressive behavior	56.4 (7.5)	54.6 (5.6)	1.27	0.21
Depressive problems	58.5 (7.1)	55.9 (6.6)	1.80	0.08
Anxiety problems	58.1 (7.6)	56.1 (7.0)	1.30	0.20

Internalizing problems	56.5 (10.3)	53.3 (10.0)	1.51	0.13
Externalizing problems	52.5 (10.1)	50.0 (9.1)	1.24	0.22
Total problems	57.6 (9.6)	53.1 (9.5)	2.26	0.026

Abbreviation: YSR = Youth Self‐Report.

### 3.4 Associations Between Stress and Psychological Difficulties

To understand the relation between lifetime stressor exposure and psychological difficulties in HD at‐risk participants and controls, we performed bivariate Spearman correlations between the STRAIN subscales and YSR subscales of interest (Table 4). In the at‐risk participants, total lifetime stressor count was significantly correlated with rule‐breaking behavior (*r* = 0.74, *p* < 0.001), depressive problems (*r* = 0.52, *p* < 0.001), and externalizing problems (*r* = 0.62, *p* < 0.001), while total lifetime stressor severity was significantly associated with rule‐breaking behavior (*r* = 0.68, *p* < 0.001), aggressive behavior (*r* = 0.45, *p* = 0.003), depressive problems (*r* = 0.55, *p* < 0.001), and externalizing problems (*r* = 0.63, *p* < 0.001), after correction for multiple comparisons.

**TABLE 4:** **Bivariate correlations in at‐risk participants and controls**. In at‐risk individuals, total lifetime stressor count was significantly related to depressive problems, rule‐breaking behavior, and externalizing problems, and total lifetime stressor severity was associated with depressive problems, after correcting for multiple comparisons. In controls, total lifetime stressor severity was significantly associated with anxiety problems and internalizing problems.

	Externalizing problems	Rule‐breaking behavior	Aggressive behavior	Internalizing problems	Anxiety problems	Depressive problems
**At‐risk**						
Total lifetime stressor count	0.62^***^ ^†^	0.74^***^	0.44^**^	0.42^**^	0.20	0.52^***^
Total lifetime stressor severity	0.63^***^	0.68^***^	0.45^**^	**0**.40^**^	0.22	0.55^***^
**Control**						
Total lifetime stressor count	0.31^*^	0.31^*^	0.22	0.38^**^	0.37^**^	0.33^*^
Total lifetime stressor severity	0.27^*^	0.27	0.19	0.47^***^	0.49^***^	**0**.37^**^

***p < 0.001, **p < 0.01, and *p < 0.05.

^†^ Bold text indicates significance after correction for multiple comparisons.

In the control group, only the correlations between total lifetime stressor severity and anxiety problems (*r* = 0.49, *p* < 0.001) and internalizing problems (*r* = 0.47, *p* < 0.001) remained significant after correction for multiple comparisons.

Fisher's *Z*‐tests demonstrated that the correlations between lifetime stressor exposure and rule‐breaking behavior were significantly stronger in at‐risk participants compared to controls (total lifetime stressor count: *z* = 2.92, *p* = 0.004; total lifetime stressor severity: *z* = 2.56, *p* = 0.01). Similarly, the correlation between lifetime stressor severity and externalizing problems was significantly stronger in at‐risk participants compared to controls (*z* = 2.15, *p* = 0.03). The difference in correlation coefficients for total lifetime stressor count and externalizing problems approached significance (*z* = 1.87, *p* = 0.061).

We next performed linear regression analyses to examine demographic characteristics and lifetime stressor exposure as predictors of internalizing problems, externalizing problems, rule‐breaking behavior, and aggressive behavior in both at‐risk individuals and controls. After adjusting for age, gender, and income, total lifetime stressor count was significantly associated with scores on each psychological symptom scale (Table 5). Group × STRAIN interaction terms were initially included in these regression models but were non‐significant and therefore excluded from the final analyses.

**TABLE 5:** Linear regression analyses predicting psychological symptoms in at‐risk individuals and controls. After adjusting for age, gender, and income, total lifetime stressor count was significantly associated with scores on each psychological symptom scale.

	Internalizing problems		Externalizing problems	
	*b*	*β*	*b*	*β*
	*F* = 4.05^**^, *R^2^* = 0.19		*F* = 5.07^***^, *R^2^* = 0.22	
Intercept	56.07^***^	—	46.84^***^	—
Age	−0.38	−0.10	−0.09	−0.02
Gender	2.15	0.11	0.23	0.01
Income	0.00	0.01	0.00	0.15
Group	0.11	0.01	0.88	0.05
Total lifetime stressor count	0.28	0.46^***^	0.29	0.49^***^

	Rule‐breaking behavior		Aggressive behavior	
	*b*	*β*	*b*	*β*
	*F* = 7.64^***^, *R^2^* = 0.30		*F* = 3.01^*^, *R^2^* = 0.15	
Intercept	50.02^***^	—	57.12^***^	—
Age	0.22	0.11	−0.44	−0.17
Gender	−0.99	−0.10	0.06	0.01
Income	0.00	0.13	0.00	0.17
Group	0.34	0.03	1.39	0.11
Total lifetime stressor count	0.15	0.48^***^	0.17	0.43^***^

***p < 0.001, **p < 0.01, and *p < 0.05.

Linear regression analyses by group showed significant associations between total lifetime stressor count and rule‐breaking behavior for at‐risk participants (*R* ^2^ = 0.40, *p* < 0.001) but not controls (*R* ^2^ = 0.17, *p* = 0.05), after adjusting for age, gender, and income. Associations between total lifetime stressor count and externalizing problems were also significant for at‐risk individuals (*R* ^2^ = 0.34, *p* = 0.004) but not controls (*R* ^2^ = 0.14, *p* = 0.11).

## 4 Discussion

In this study, we compared lifetime stressor exposure in adolescents at risk for HD and controls and examined how stressors are related to psychological difficulties in these cohorts. Our results show that at‐risk individuals experience more acute and chronic stressors and report greater severity of stressors than their peers. In particular, youth at risk for HD report significantly more stressors involving Treatment/Health and Role Change/Disruption. Interestingly, while stress exposure was primarily related to anxiety problems and internalizing problems in controls, greater stressor exposure was instead significantly associated with rule‐breaking behavior, externalizing problems, and depressive problems in at‐risk individuals.

Together, these findings provide novel insights into the breadth and characteristics of stressors faced by youth at risk for HD across the life course. While previous studies have focused on brief self‐report checklists of stressors, the STRAIN provides a much more comprehensive assessment of stressors spanning multiple life domains and social‐psychological characteristics, increasing its sensitivity to detect a wider range of potential sources of stress. Moreover, with a mean time‐to‐completion of 27 minutes and no reported complaints from participants, the STRAIN was usable and acceptable in our study population.

The quantity and breadth of stressors endorsed by our participants indicate that this instrument was able to capture relevant sources of stress for both at‐risk individuals and controls, in addition to revealing some key differences between the groups. Given their developmental age, education‐related stressors were the most frequently endorsed in both groups, while few participants in either group endorsed stressors related to work or reproduction, which is not unexpected in these two samples of adolescents. Notably, at‐risk participants endorsed greater stress than controls in 4 of 12 life domains and 5 out of 5 social‐psychological characteristics, whereas controls did not score higher in any area. In particular, at‐risk participants reported significantly more stressors related to Treatment/Health and Role Change/Disruption. These findings are consistent with observations from prior studies investigating young people's experiences growing up in families impacted by HD (Downing et al., 2012; Forrest Keenan et al., 2007; Vamos et al., 2007). However, the quantitative nature of our assessment, younger age range, and inclusion of a community control group for direct comparison with peers provide an important new perspective on sources of stress and their association with psychological difficulties in youth at risk for HD.

Interestingly, although there were no group mean differences in externalizing problems, at‐risk participants reported significantly more of these symptoms, particularly rule‐breaking behavior, when exposed to greater lifetime stressors. This finding suggests that youth at risk for HD may have an increased susceptibility to externalizing behaviors that is activated by exposure to stressors. This potential association between stressor exposure and rule‐breaking behavior is notable given prior work demonstrating a high prevalence of risk‐taking behavior and legal issues in patients with HD (McDonell et al., 2020, 2021). which has not yet been investigated in youth at risk for the disease. Stressor exposure has been linked to engagement in risky behaviors in adolescents undergoing mental health treatment (Slavich et al., 2019), and additional studies have shown that risk‐taking and impulsive behaviors may be related to impairments in self‐regulation and inhibitory control, increased emotional reactivity to trauma, and impaired coping skills (Bounoua and Sadeh, 2022; Bresin, 2020; Halliburton et al., 2024; Stumps et al., 2024).

Furthermore, recent work has demonstrated that adolescent and young adult offspring of parents with HD have impairments in working memory, which are associated with reduced coping skills and elevated levels of anxiety and depression in the offspring of HD‐affected parents (Ciriegio et al., 2020). Conversely, higher inhibitory control skills are associated with improvements in coping and lower rates of anxiety and depression (Ciriegio et al., 2022). Cognitive behavioral therapies to support the development of effective coping skills and interventions to support inhibitory control and working memory may therefore be potential treatment avenues to enhance young people's ability to cope with stress. Despite evidence of benefit in other juvenile chronic conditions (Kashikar‐Zuck et al., 2013; Novak, 2025), psychological interventions, including cognitive behavioral therapy, are notably lacking in the HD population as a whole and especially in children (Zarotti et al., 2020). One trial is currently underway examining a guided self‐help intervention for adult HD gene expansion carriers based on cognitive behavioral therapy and acceptance and commitment therapy (Dale et al., 2023), but more are urgently needed.

### 4.1 Strengths and Limitations

Strengths of this study included the use of well‐validated measures to explore stress and psychological difficulties in an understudied population. Importantly, our study focused on a younger age range than most previous studies of stress in HD. The fact that young people under the age of 18 have thus far been excluded from nearly all major longitudinal studies in HD has led to a significant knowledge gap regarding the experiences of youth and adolescents from HD families. This impairs our ability to provide evidence‐based support to young people at risk for HD and also limits our understanding of potential early disease manifestations.

Given that the majority of our cohort were under the age of 18, the HD genotype was not taken into account for this study. All participants have provided a saliva sample for triple‐blinded genetic testing (Pfalzer et al., 2023), which will allow for future investigations with a larger sample size to disentangle environmental effects from genetic effects. However, the extremely high prevalence of stress in our cohort and the notable differences between at‐risk youth and controls without considering genotype indicate that youth from HD‐affected families are growing up in markedly different circumstances than their peers. These findings also have important implications for selecting control groups for longitudinal studies, as community controls and family controls are likely not equivalent.

Limitations include the single‐site design, leading to greater homogeneity in our study population, as well as potential recruitment bias, given that participants were drawn from HD families already receiving clinical care or participating in research. These factors may limit generalizability to the broader HD community and may importantly overlook people who are experiencing psychological difficulties and are not able to attend regular appointments.

In this study, differences between groups on the STRAIN subscales were associated with moderate to large effect sizes (Cohen's *d* = 0.64–0.75), while group differences on ASEBA subscales were associated with smaller effect sizes (Cohen's *d* = 0.2–0.56). A larger sample would allow us to explore these relationships in more depth and would also allow for the assessment of genotype in the at‐risk group.

Additional limitations include discrepancies in ethnicity and income level between at‐risk participants and controls. HD is more prevalent in people of European descent (Pringsheim et al., 2012), but particularly in light of recent work showing more severe clinical presentations at baseline in Black patients (Buchanan et al., 2023), future studies should include a more diverse sample. The difference in median income between our groups is notable and highlights the socioeconomic disparities that families impacted by HD face. HD negatively affects employment and working capacity, even before the onset of motor symptoms (van der Zwaan et al., 2022), and the overall prevalence of HD tends to be higher in those with lower incomes (Bruzelius et al., 2019; Madera et al., 2025). A recent review found that 37% of patients with HD were single or divorced, 39% had less than 12 years of education, and 20% were uninsured or on low‐income health insurance (Pfalzer et al., 2021). These environmental factors should be considered in future studies assessing psychological and behavioral changes in youth at risk for HD.

As this is the first study to use the STRAIN in an HD cohort, future studies should further evaluate its feasibility and applicability in the broader HD population. Investigating the associations between stress and psychological difficulties in a larger sample will also allow for the incorporation of blinded genetic testing data to distinguish environmental vs. genetic effects. Longitudinal follow‐up will also help to determine the directionality of these relationships and to investigate whether stress predicts future psychological difficulties.

## 5 Conclusion

In summary, this study provides new insights into the frequency and characteristics of lifetime stressor exposure in adolescents at risk for HD using a comprehensive interview‐based measure. The data also show a significant relationship between lifetime stressor exposure and externalizing problems in at‐risk youth. These findings thus emphasize the need for evidence‐based protocols to support young people in HD families and highlight the importance of considering stress and environmental factors in the assessment of early behavioral changes in HD.

## Author Contributions

**Katherine E. McDonell**: Conceptualization, Data Curation, Formal Analysis, Funding Acquisition, Investigation, Writing – original draft, Writing – review and editing; **Shuhei Shiino**: Data Curation, Investigation, Project Administration, Writing – review and editing; **Elizabeth M. Key**: Data Curation, Investigation, Project Administration, Writing – review and editing; **Louis DeLuna**: Data Curation, Investigation, Project Administration, Writing – review and editing; **Kelly H. Watson**: Conceptualization, Writing – review and editing; **Bruce E. Compas**: Conceptualization, Supervision, Writing – review and editing, **George M. Slavich**: Resources, Software, Writing – review and editing; **Daniel O. Claassen**: Conceptualization, Supervision, Writing – review and editing.

## Ethics Statement

This study was reviewed and approved by the Vanderbilt Institutional Review Board (IRB # 220196).

## Consent

All participants under the age of 18 completed a written assent form, and a parent or legal guardian provided written informed consent. Participants over the age of 18 provided written informed consent.

## Conflicts of Interest

The authors declare no conflicts of interest.

## Data Availability

Deidentified data that support the findings of this study are available from the corresponding author upon reasonable request.
